# Reply to: Analysis of covariance and sample size calculation for comparing means in randomized controlled trials

**DOI:** 10.34172/jcvtr.2023.33034

**Published:** 2023-12-30

**Authors:** Naimeh Mesri Alamdari, Samad Ghaffari, Neda Roshanravan

**Affiliations:** ^1^Endocrine Research Center, Tabriz University of Medical Sciences, Tabriz, Iran; ^2^Cardiovascular Research Center, Tabriz University of Medical Sciences, Tabriz, Iran

## The Authors Reply

 We profoundly thank Alimohamadi et al^1^ for their great attention and for carefully evaluating the clinical trial “Effects of sodium selenite and selenium-enriched yeast on cardiometabolic indices of patients with atherosclerosis: A double-blind randomized clinical trial study”.^2^ Alimohamadi et al^1^ have proposed some concerns that are better to be discussed here. The answers to the questions are in the order that have been raised.

 The first concern is about the determination of sample size. It should be noted that the calculation of sample size is based on the mean change ( ± standard deviation [SD]) of GPX in Hu et al^3^ study. We consider the second group’s mean ( ± standard deviation [SD]) with 20% changes relative to the other group and they were put in the following formula:


$$N=\frac{2{\left(Z_{1-\alpha /2}+Z_{1-\beta }\right)}^{2}S_{p}^{2}}{\delta^{2}}$$

Thus, the calculation of sample size is per group and there are between-group comparisons. So, implicitly, we multiplied the calculated sample size by 3 which is much bigger than [sqrt (3)-1]. Unfortunately, it was not clearly explained in the paper. However, the method of determination of sample size was properly done as mentioned by Alimohamadi et al^1^.

 Regarding the second concern about the statistical analysis, it should be indicated that we have analyzed data based on mean differences reports. Since the distribution of data was not normal and we were not allowed to use the analysis of covariance (ANCOVA) test, we calculated the difference and used the Kruskal-Wallis Test and compare change between groups. Noted that none of transformations (log, square, inverse,...) were effective to transform data into normal. It is important to emphasize that there is no fault in content and all the analysis methods are properly done. However, it is not completely well-reported in some parts which may confuse the readers.

## Competing Interests

 None declared by the authors.

## Funding

 None.
